# Comparison of oral dydrogesterone with vaginal progesteronefor luteal support in IUI cycles: a randomized clinical trial

**Published:** 2015-07

**Authors:** Donya Khosravi, Robabeh Taheripanah, Anahita Taheripanah, Vahid Tarighat Monfared, Seyed-Mostafa Hosseini-Zijoud

**Affiliations:** 1 *Infertility and Reproductive Health Research Center (IRHRC), Shahid Beheshti University of Medical Sciences, Tehran, Iran.*; 2 *Department of Molecular and Cellular Sciences, Faculty of Advanced Sciences and Technology Pharmaceutical Sciences Branch, Islamic Azad University, Tehran-Iran.(IAUPS).*; 3 *Islamic Azad Univercity, Saveh Branch, Saveh-Iran.*; 4 *Social Development and Health Promotion Research Center, Kermanshah University of Medical Sciences, Kermanshah, Iran.*

**Keywords:** *Progestins*, *Luteal-phase*, *Gonadal hormones*

## Abstract

**Background:**

The aim of this study, we have compared the advantages of oral dydrogestrone with vaginal progesterone (cyclogest) for luteal support in intrauterine insemination (IUI) cycles. Progesterone supplementation is the first line treatment when luteal phase deficiency (LPD) can reasonably be assumed.

**Objective:**

This study was conduct to compare the effect of oral dydrogestrone with vaginal Cyclogest on luteal phase support in the IUI cycles.

**Materials and Methods:**

This prospective, randomized, double blind study was performed in a local infertility center from May 2013 to May 2014. It consisted of 150 infertile women younger than35years old undergoing ovarian stimulation for IUI cycles. They underwent ovarian stimulation with oral dydrogesterone (20 mg) as group A and vaginal cyclogest (400 mg) as group B in preparation for the IUI cycles. Clinical pregnancy and abortion rates, mid luteal progesterone (7daysafter IUI) and patient satisfaction were compared between two groups.

**Results:**

The mean serum progesterone levels was significantly higher in group A in comparison with group B (p=0.001). Pregnancy rates in group A was not statistically different in comparison with group B (p =0.58). Abortion rate in two groups was not statistically different (p =0.056) although rate of abortion was higher in group B in comparison with A group. Satisfaction rates were significantly higher in group A compared to group B (p<0.001).

**Conclusion:**

We concluded that oral dydrogestrone is effective as vaginal progesterone for luteal-phase support in woman undergoing IUI cycles. Moreover, the mean serum progesterone levels and satisfaction rates in dydrogestrone group were higher than cyclogest group.

## Introduction

Intrauterine insemination (IUI) is a common treatment in sub-fertile male, unexplained infertility and coital or cervical problems (1) that its utilization has increased in the recent decades because it is a simple, non-invasive, and a cost-effective technique (2). Consequently, IUI has been performed generally combined with controlled ovarian hyperstimulation (COH), i.e., with Clomiphene citrate and/or Gonadoptroin or their combination (3). There are certain variables that are known to be predictive of IUI success and luteal phase support. The average success rate is between 11-20% (4).

The luteal phase is defined as the period between the ovulation and pregnancy occurrence or starting the new menstruation (5). Ovulation induction with the change in endocrine metabolism has negative effect on the luteal phase function (5). The luteal phase deficiency was first described by Jones in 1949 (6). The reported prevalence of luteal phase deficiency (LPD) ranges from 3.7% to 20% among patients with infertility (6). Luteal support was not performed routinely in all IUI cycles. It is recommended as the luteal phase support for the cycles with the mid-luteal progesterone was < 10 ng/mL (5). Ovulation induction with growth of many follicles induces the hyeprestrogenemic state that cannot compensate with progesterone. So, the it seems that luteal phase deficiency is higher in induction ovulation cycles with or without IUI. 

Although there are many protocols for controlled ovarian stimulation but there isn't any accepted opinion about the best appropriate regimen for luteal phase support. Recent studies have been shown that luteal phase support improved the success of IUI cycles affecting both clinical pregnancy and live birth rates (5, 7-8).

Currently, progesterone supplementation is the first line treatment when LPD can reasonably be assumed (5, 9). Progesterone induces a secretory transformation of the uterine glands, increases vascularity of the endometrial lining, and stabilizes the endometrium in preparation for embryo implantation (10). Also, progesterone potentially sustains the survival of the embryo by shifting the immune system toward production of non-inflammatory T-helper (Th) 2 cytokines (11). After choosing progesterone as a therapeutic option, one must then ascertain the optimal form, dosage, and timing to use for each individual. Available products include both "synthetic" and "natural" progesterone. Synthetic progesterone are not as quickly processed or eliminated by the body, so their activity is prolonged. Progesterone can be administered orally, vaginally, or through intramuscular(IM) injection (12). The anatomy of vagina with its rich vascular plexus provides an ideal environment for absorbing drugs. The rugae of the vaginal wall increase the total available surface area (13). Vaginal administration results in higher uterine concentrations, but is often uncomfortable in the presence of vaginal bleeding, or may be washed out if bleeding is severe (14). Oral dosing requires a higher concentration in order to compensate for "first-pass" liver metabolism (15), but oral administration is the easiest route of administration, and generally the most acceptable route for the patient (14). On the other hand, it seems that dydrogesterone has the immunologic effects and it is associated with higher rate of pregnancy and even lower pregnancy complications such as fetal distress and gestational hypertension (16). Therefore, the aim of this prospective study was to compare the effect of oral dydrogesterone with vaginal progesterone as the luteal phase support on the outcome of IUI cycles.

## Materials and methods

**Fig. 1:**
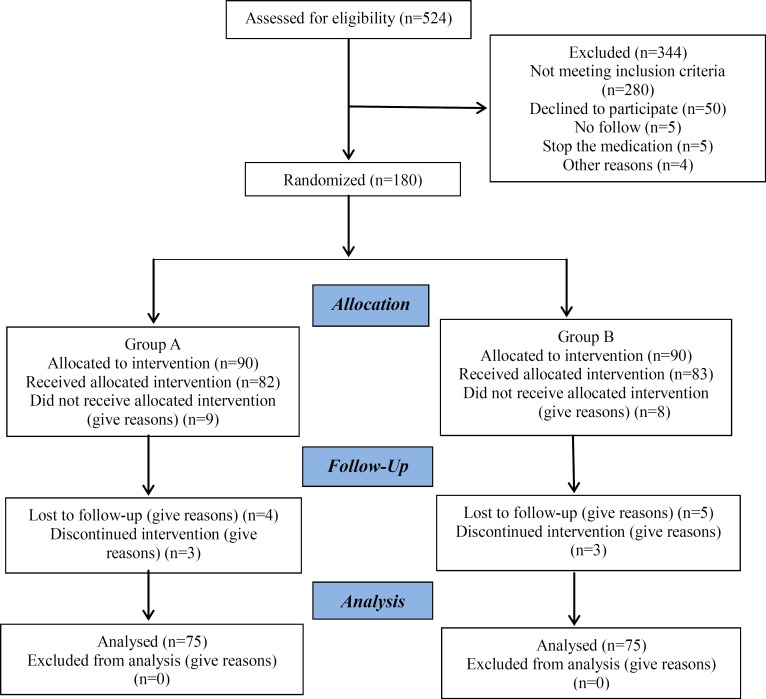
The flowchartoftheallocationofpatientsintotwogroupsandfollowedthem

In this prospective randomized clinical trial, 524 patients that they were candidated for IUI were enrolled. But 344 couples were excluded due to in -cooperation, and other given reasons. At the end, we analyzed 180 unexplained infertility women who underwent ovulation induction and intrauterine insemination (IUI) between May 2013 and August 2014 at infertility and reproductive Health research center and Emam Hossein Hospital, Tehran. They were divided randomly into two groups according to the based on a computer generated list, while neither the patients nor the procedure developer had any information about the treatment assignment. (Fig.1). Group A: 90 patient for the oral progesterone and the second group (Group B) was consisted of 90 patients for vaginal progesterone.

Before selecting the patient for treatment, all of them underwent the following tests: hysterosalpingography, basal FSH, LH and AMH hormone concentrations of third day of mensturation, semen analysis. Inclusion criteria were: age <35 years, normal hormonal assay, normal pelvis in transvaginal sonography, duration of infertility ≤ 5 years, and bilateral tubal patency at hysterosalpingography. Exclusion criteria were: Basal levels of follicle stimulating hormone (FSH) ≥10mlU/ml, High grade endometriosis stage, or a history of abdominal surgery or severe male factor infertility. A written informed consent was obtained from each couple. This study was approved by the ethical committee of Shahid Beheshti medical university. The ovarian stimulation protocol was consisted of clomiphene citrate (Clomid, serophen, Iran Hormone) 100mg daily for 5 days starting from day3, Then treatment was continued with gonadotropin (FSH recombinant, Gonal-F, Serono, Switzerland) 150 IU from 8th day of cycleuntil at least 2-3 follicles reached to 18-20 mm in diameter. Then 10000 IU of human chorionic gonadotropin (Choriomon, IBSA, Switzerland) was injected intramuscularly for triggering of ovulation. Intrauterine insemination with washed sperm was done 36 -38 hours after hCG injection. 8 patients in group A and 7 people from the Group B were excluded from the study because of the ovarian hyper stimulation syndrome. Luteal phase support was started 48 hours after IUI with10 mg dydrogesterone twice per day in group A versus 400 mg vaginal progesterone once per night in group B. serum progesterone level was measured on the mid-luteal phase, 7 days after IUI by the kit of Monobind (ELISA). Progesterone administration was continued for 2 weeks. If the BHCG was positive then the medication were continued till 10 weeks of pregnancy. 7 patients in the group A and 8 patients in the group B were excluded from the study due to lost to follow up or discontinuation of the treatment. Clinical pregnancy rates, abortion rates, serum mid luteal progesterone and patient satisfaction in both groups were compared in the final 75 patients in each group.

**Statistical analysis**

All statistical analyses were done using SPSS 20.0 (Statistical Package for the Social Sciences, version 20.0, SPSS Inc, Chicago, Illinois, USA). Continuous and categorical variables were compared with T-test or χ2 test, respectively. Results are reported as mean value ± standard deviation and categorical values were expressed in relative frequency. The P value of < 0.05 was considered as statistically significant.

## Results

In this research, 180 patients were studied who were divided into two groups (A received oral dydrogesterone and B received vaginal cyclogest). There were 90 patients in each group. The mean age of woman in group A and B was 30.9±3.9 and 30.5±4.0years old, respectively. The mean age of male partner in group A and B was 35.3±5.6 and 34.9±6.1 years old, respectively. The mean number of follicles was 3.5±1.7 in group A and 3.4±1.8 in group B. (P= 0.076). Two groups were matched regarding to their age, partner age, and the number of follicles duration of infertility, serum FSH in 3th day of cycle and sperm quality (Table I).

Serum progesterone levels were significantly different between two groups (P=0.001). The mean serum progesterone levels in group A (dydrogesterone) was higher than group B (Cyclogest) (Table II). The results showed that the two drugs were equally effective infertility (p=0.58). 29.7% of patients, who received dydrogesterone, were pregnant and fertility rate was 25.7% in patients who received cyclogest (Table II). Although, abortion rate was higher in women who received cyclogest compared to dydrogesterone group but this difference was not statistically significant (p=0.056) (Table II). The patient satisfaction rates were significantly higher in group A than to group B (p<0.001).

**Table I:** Baseline patient's characteristics

**Variables**		**Group A (Dydrogesterone)**	**Group B (vaginal progesterone)**	**p value**
**Age ** **(years)** ** (mean±SD)**		30.9±3.9	30.5±4	0.578
**Husband age ** **(years) ** **(mean±SD)**		35.3±5.6	34.9±6.1	0.065
**Duration of infertility (years)**		4.1±2.6	3.5±2.4	0.114
**FSH day 3 (IU/L) **		6.1±2.9	5.4±2.5	0.131
**Number of follicles**		3.5±1.7	3.4±1.8	0.076
**Spermquality (%)**	**Normal**	86.5	89.2	0.616
	**Abnormal**	13.5	10.8	0.123

Variables were compared withT-test or χ2 test.

P value of < 0.05 was considered as statistically significant.

**Table II:** Clinical outcome of patients undergoing treatment with Dydrogesterone (group A) and with Cyclogest (group B)

**Variables**		**Group A (Dydrogesterone)**	**Group B (Cyclogest)**	**p value**
**Serum levelsof progesterone (mean±SD)**		52.6±29.9	28.9±159	0.001
**Pregnancy rates.No (%)**		22 (29.7)	19 (25.7)	0.582
**Abortion rates.No (%)**		2 (9.1)	3 (15.8)	0.056
**Patientsatisfaction. No (%)**	**Yes**	63 (85.1)	46 (60.8)	0.001
	**No**	12 (14.9)	29 (39.2)	
		

Variables were compared withT-test or χ2 test.

P value of < 0.05 was considered as statistically significant.

## Discussion

In the recent years impressive advances have been occurred in the treatment of infertility and assisted reproductive techniques. But more simple treatments like IUI has been important treatment for the subfertile couples (16). The final goal of this treatment is to achieve a pregnancy and deliver a healthy live baby. The probability of pregnancy with IUI depends on various factors including age of the couple, type of sub-fertility, ovarian stimulation and the luteal phase support (17-21). As it is not known whether the luteal phase deficiency is compromised the IUI results or not, many physicians recommend the support of this phase with progesterone.

Progesterone prepares the endometrium for pregnancy which is produced by the corpus luteum. This occurs in the luteal phase of the menstrual cycle (22) but in stimulated cycles it is compromised due to hormonal imbalance and hyperestrogenemic state. Progesterone supplementation is the most commonly used treatment in IUI cycles and it is a logical step to improve the chance of success (23, 24). Dydrogestrone is a retroprogesterone with good oral bioavailability that has a biological active metabolite of progesterone (25). On the other hand some studies have shown that dydrogesterone with systematic effects on the immunological factors may improve the implantation rate and decrease the abortion rate.

In this study, we compared the advantages of oral dydrogestrone with vaginal progesterone (cyclogest) for luteal support in IUI cycles. According to the results of this study, the mean serum progesterone levels in group A (Dydrogesterone) was higher than group B (Cyclogest) (p=0.001). Conversely Levine et al compared the pharmacokinetics of an oral micronized progesterone preparation with that of a vaginal progesterone gel and showed that the vaginal gel was associated with a higher maximum serum concentration of progesterone. They concluded that the vaginal administration of progesterone results in a greater bioavailability with less relative variability than oral progesterone (26). On the other hand some studies have shown that there is no significant improvement in the pregnancy rate with luteal phase support in comparison with unsupported cycles in IUI (4, 27). Luteal phase support is more useful in IUI or induction ovulation cycles with gonadoptroin and does not have any effect on clomiphene citrate. There is an unknown mechanism for the potential difference in endogenous luteal phase function depending on the method of ovulation induction that causes no effect of luteal phase support in clomiphene citrate versus of gonadotropin stimulated cycles with or without IUI (3, 28).

Our results showed that abortion happened higher in vaginal group in comparison with dydrogestrone group and this difference was not statistically significant (p>0.05). Crap et al. evaluated the effects of dydrogesterone on abortion rates in infertile women. There was a 13% (44/335) miscarriage rate after dydrogesterone administration compared to 24% in control women [odds ratio for miscarriage 0.47, (CI=0.31-0.7), 11% absolute reduction in the miscarriage rate] (25). Fatemiet al. announced that after estrogen endometrial priming in POF patients, exogenous vaginal micronized progesterone is more effective than oral dydrogesteronein creating an ‘in-phase' secretory endometrium and induces significantly higher progesterone and lower LH and FSH serum concentrations on day 21 of the cycle (29). Ganesh et al. compared oral dydrogestrone with progesterone gel and micronized progesterone for luteal-phase support and indicated no significant difference among three groups of women regarding the overall pregnancy and miscarriage rate (30). Patkiet al. indicated that the pregnancy rate is significantly higher with dydrogesterone than with micronized vaginal progesterone and placebo (31).

The results of our study showed that satisfaction rates were significantly higher in group A (who received Dydrogesterone) compared to group B (who received Cyclogest) (p=0.001). Chakravarty et al. in a prospective, randomized study compared the efficacy, safety and tolerability of vaginal micronized progesterone with oral dydrogesterone as luteal phase support after in-vitro fertilization (IVF). The results of their studies indicated that more patients given dydrogesterone than micronized progesterone were significantly satisfied with the tolerability of their treatment (p<0.05) (32).

## Conclusion

We showed that oral dydrogestrone is as effective as vaginal progesterone for luteal-phase support in woman undergoing IUI. Moreover, the mean serum progesterone levels and satisfaction rates in dydrogestrone group were higher than cyclogest group.
